# Graphene oxide electrochemistry: the electrochemistry of graphene oxide modified electrodes reveals coverage dependent beneficial electrocatalysis

**DOI:** 10.1098/rsos.171128

**Published:** 2017-11-15

**Authors:** Dale A. C. Brownson, Graham C. Smith, Craig E. Banks

**Affiliations:** 1 Faculty of Science and Engineering, Manchester Metropolitan University, Chester Street, Manchester M1 5GD, UK; 2 Faculty of Science and Engineering, Department of Natural Sciences, University of Chester, Thornton Science Park, Pool Lane, Ince, Chester CH2 4NU, UK

**Keywords:** graphene oxide, graphene, highly ordered pyrolytic graphite, electroanalysis, electron transfer, electrochemistry

## Abstract

The modification of electrode surfaces is widely implemented in order to try and improve electron transfer kinetics and surface interactions, most recently using graphene related materials. Currently, the use of ‘as is’ graphene oxide (GO) has been largely overlooked, with the vast majority of researchers choosing to reduce GO to graphene or use it as part of a composite electrode. In this paper, ‘as is’ GO is explored and electrochemically characterized using a range of electrochemical redox probes, namely potassium ferrocyanide(II), N,N,N′,N′-tetramethyl-p-phenylenediamine (TMPD), dopamine hydrochloride and epinephrine. Furthermore, the electroanalytical efficacy of GO is explored towards the sensing of dopamine hydrochloride and epinephrine via cyclic voltammetry. The electrochemical response of GO is benchmarked against pristine graphene and edge plane-/basal plane pyrolytic graphite (EPPG and BPPG respectively) alternatives, where the GO shows an enhanced electrochemical/electroanalytical response. When using GO as an electrode material, the electrochemical response of the analytes studied herein deviate from that expected and exhibit altered electrochemical responses. The oxygenated species encompassing GO strongly influence and dominate the observed voltammetry, which is crucially coverage dependent. GO electrocatalysis is observed, which is attributed to the presence of beneficial oxygenated species dictating the response in specific cases, demonstrating potential for advantageous electroanalysis to be realized. Note however, that crucial coverage based regions are observed at GO modified electrodes, owing to the synergy of edge plane sites and oxygenated species. We report the true beneficial electrochemistry of GO, which has enormous potential to be beneficially used in various electrochemical applications ‘as is’ rather than be simply used as a precursor to making graphene and is truly a fascinating member of the graphene family.

## 1. Introduction

Carbon materials have been widely used in both analytical and industrial electrochemistry for a considerable number of years [1,2]. In addition to using an electrode comprised entirely of the desired carbon material, modification of various electrode substrates with differing carbon materials is readily performed with the aim of enhancing the electrochemical characteristics and performance of the fabricated electrode [3,4]. Most recently, graphitic nanomaterials have been at the forefront of innovative research, with enormous interest from electrochemists focusing on graphene because of its reported extraordinary physical, chemical and electrical properties [5]. Graphene has been reported to exhibit advantageous behaviour for a wide variety of electrochemical applications [5–8], however, it must be noted that fundamental reports have emerged which indicate that in certain aspects of graphene electrochemistry it may not be such a beneficial electrode material as first thought [9–11].

The fundamental understanding of electron transfer at graphitic electrode materials indicates that they are electronically anisotropic in nature [2,11,12]. It is widely acknowledged that the observed electron transfer at graphene originates from two heterogeneous structural contributions; namely the edge plane- and basal plane-like sites/defects [11], where the heterogeneous electron transfer (HET) rate of the former (its peripheral edge, or at edge defect sites) is anomalously faster over that of the latter (its side/face), which in comparison can be regarded as relatively electrochemically inert [13–15]. What makes the electrochemistry of graphitic materials highly fascinating is that various forms (*viz.* graphite, graphene, carbon nanotubes (CNTs), etc.) display differing electrochemical characteristics owing to their distinct morphologies (different ratios of edge to basal plane content) [11,16,17]. This distinction gives rise to unique electronic properties (varied Density of States; DoS) that dictate the electrochemical responses observed, where it has been shown that a material with a low coverage of edge plane sites (i.e. pristine graphene) resultantly exhibits unfavourable electronic properties [9,11]. However, improvements or variations in the DoS can be induced by the respective presence (or absence) of specific impurities (both those inherent to the fabrication process and those purposely incorporated, such as through chemical doping), functionalisation of the carbon structures, or through the synthesis of composite materials[18–22]: these factors also lead to alterations in the specific surface interactions that occur at carbonaceous materials [2,11]. Most notably, the presence of specific oxygenated functionalities has been shown to strongly influence surface interactions which consequently results in significant changes in the observed voltammetry either beneficially or detrimentally and have the potential to be used favourably within a multitude of electrochemical applications [23–26].

Graphene oxide (GO) comprises a single atomic layer of functionalized (oxygenated) graphene, thus makes for an interesting material to study in electrochemistry owing to expected contributions in the observed voltammetry arising from edge/basal plane sites as well as from the oxygenated species present [11,27]. GO is by no means a new material given that it has been known to exist since the 1840s [28]; however, until now it has been largely overlooked in this field of research, being considered predominantly as a precursor for graphene synthesis [11,29]. Although GO has been reported to be beneficial in a number of technological areas within electrochemistry [30,31], such as in the fabrication of energy storage devices [32], implementation as a membrane material [33], the monitoring of nucleic acids [34] and decoration with platinum to simultaneously characterize ascorbic acid, dopamine and uric acid levels [35], it is apparent from the literature that in the majority of cases reduced GO is used or GO is incorporated as part of a hybrid/composite material rather than using it ‘*as is*’ [11,27,36,37]. This is because GO is reported in the literature to be an insulator [38], suggesting it is not as useful as its counterpart graphene. As such, the basic voltammetric understanding of GO is clearly lacking within the literature and the electrochemical properties of this intriguing and potentially beneficial material have yet to be fully characterized.

The focus herein is to electrochemically characterize GO modified electrodes with both inner- and outer- sphere redox probes, before exploring its application as an electrocatalytic sensor substrate towards the detection of electroactive biological analytes that are of high importance within the literature. We reveal interesting insights into the fundamental knowledge of carbonaceous materials, where owing to GO's mixture of edge plane sites/defects and abundant oxygenated species, unique voltammetry is observed and reported, to our knowledge for the first time. We demonstrate that these unique voltammetric signatures, observable only with GO, are coverage dependent.

## 2. Experimental section

All chemicals were of analytical grade (or higher) and were used as received from Sigma-Aldrich without any further purification. All solutions were prepared with deionised water of resistivity not less than 18.2 MΩ cm and were vigorously degassed prior to electrochemical measurements using high purity, oxygen free nitrogen.

The voltammetric measurements were recorded using an ‘Autolab PGSTAT 101’ (Metrohm Autolab, The Netherlands) computer-controlled potentiostat. All measurements were conducted using a three-electrode system. The edge plane-pyrolytic graphite (EPPG) working electrode (Le Carbone, Ltd. Sussex, UK) was machined into a 4.9 mm diameter with the disc face parallel to the edge plane as required from a slab of HOPG (highest grade available: SPI-1, equivalent to Union Carbide's ZYA grade, with a lateral grain size, *L_a_* of 1–10 µm and 0.4 ± 0.1° mosaic spread). Alternatively, the basal plane-pyrolytic graphite (BPPG) working electrode was machined as per the EPPG however with the disc face parallel with the basal plane as required. A platinum wire and a saturated calomel electrode (SCE) were used as counter and reference electrodes respectively.

Commercially available GO was purchased from ‘Graphene Supermarket’ (Reading, MA, USA) [39] and consists of ‘single layered GO dispersed in water’ at a concentration of 275 mg l^–1^. Pristine graphene was commercially obtained from ‘Graphene Supermarket’ [39] and are known as ‘Pristine Graphene Monolayer Flakes’ comprising entirely of pristine graphene platelets dispersed in ethanol (1 mg l^–1^) that have not been oxidized, reduced or chemically modified in anyway and are free from surfactants. Further details of the GO and graphene used in this work are available in the electronic supplementary material (figures S1–S4); this includes details on their fabrication and full physical and chemical characterization. In summary, this shows that the GO has an average flake size of between 0.5 and 5.0 micrometres and a thickness of 1 atomic layer. Independent Raman spectroscopy, transmission electron microscopy (TEM), X-ray photoelectron spectroscopy (XPS) and X-ray diffraction (XRD) analysis confirm the presence of high quality/purity GO by structural characterization, with the XPS chemical analysis indicating the material to comprise 66.8% atomic carbon and 28.6% atomic oxygen. Specifically, groups corresponding to graphitic C─C bonding in addition to C─O or C─O─C bonds (47.21%, 286.7 eV) and C═O or COO (7.94%, 288.4 eV) bonds where characteristically present, which is in excellent agreement with previous literature reports regarding GO [11,36,40,41]. The graphene has an average flake thickness of 0.35 nm (1 monolayer) with an average particle (lateral) size of 550 nm (150–3000 nm). Independent TEM and Raman spectroscopy confirms monolayer graphene is present with little/no defects and XPS chemical analyses indicate the material to comprise 95.84% atomic carbon and 4.16% atomic oxygen. The Raman interpretation and low O/C ratio suggests near true graphene is present, that is to say that we use single layered ‘pristine’ graphene sheets that possess low oxygen content and a low coverage of edge plane like- site/defects [11,12]. Furthermore, stringent electrochemical control experiments were performed to substantiate the integrity of experiential data (i.e. using ‘blank’ solutions and at solvent modified electrodes); these results can also be found in the electronic supplementary material (figure S5). Once received from the supplier, aliquots of the GO were carefully pipetted onto the electrode surface using a micropipette and dried under heat (50°C) before being allowed to cool to ambient temperature, following which the electrode could either be further modified or was ready to use. For the case of graphene, the same procedure was adhered to with the exception of the drying step, where graphene was allowed to dry at room temperature under nitrogen flow in order to eliminate oxidation of the graphene by the presence of atmospheric oxygen.

Note that as is the case with impurities in CNTs [11], if metals such as Fe remain on the GO following its fabrication, then in such cases these metal impurities could be a source of electrochemical reactivity [11,42]. Given the wide range of fabrication routes available, with each employing distinct processes and chemicals, it is clear that depending upon the method of synthesis (and the specific metal is potentially present) the reactivity and inherent properties of the fabricated GO material can change drastically [11]. The characterization presented herein shows *clearly* that no such metals are present within our materials and thus the responses observed throughout are attributed solely to that of the GO itself.

## 3. Results and discussion

### 3.1. Electrochemical characterization

**Figure 1.**
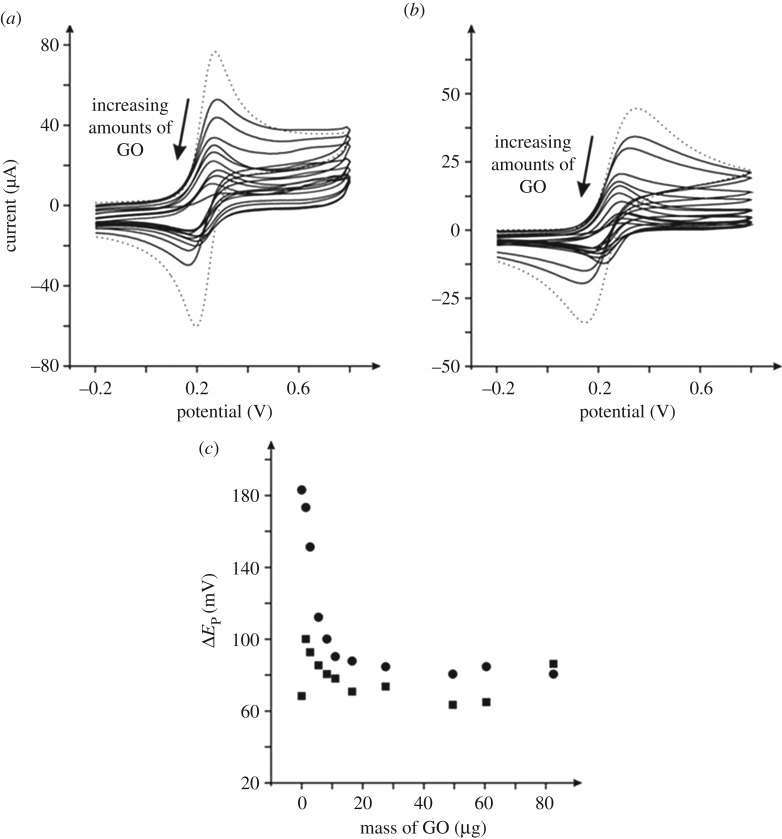
Cyclic voltammetric responses of µg amounts of GO modified EPPG (*a*), and BPPG (*b*) electrodes recorded in 1 mM ferrocyanide/0.1 M KCl. All data are obtained at a scan rate of 100 mV s^–1^ (versus SCE). The response of the unmodified/bare EPPG and BPPG is shown in (*a*) and (*b*) as the dotted lines respectively. (*c*) The analysis of the peak-to-peak separation (Δ*E*_P_) from (*a*) and (*b*) as a function of GO coverage; circles represent BPPG and squares the EPPG electrode respectively.

The electrochemical response of a bare/unmodified EPPG and a BPPG electrode was first benchmarked and characterized using the electron transfer redox probe, 1 mM potassium ferrocyanide(II)/0.1 M KCl. Figure 1 depicts cyclic voltammetric signatures of bare (unmodified) EPPG and BPPG electrodes which exhibit electrochemically characteristic signatures with peak-to-peak separations (Δ*E*_P_'s) of 68.4 and 183.1 mV respectively (at 100 mV s^–1^ versus SCE). Analysis of the voltammetric peak height as a function of the square-root of scan rate reveals a highly linear response, indicating a diffusional electrochemical process as expected and widely reported in the literature using this redox probe and electrode substrate [12]. Analysis of the peak-to-peak separation (Δ*E*_P_) indicates a dependence on voltage scan rate, indicating the electrochemical process to be classed as *quasi*-reversible within the scan rates employed. To benchmark our electrochemical system/setup, the theoretically predicted current response was deduced using the following *quasi*-reversible Randles–Ševćik equation (at 298 K) [1,43]: $I_{p,\mathrm{quasi}}=(2.65\times 10^{5})n^{3/2}AD^{1/2}C\upsilon^{1/2}$, where *D* is the diffusion coefficient of the electroactive probe ($6.5\times 10^{-6}\;{\text{cm}}^{2}\;{\text{s}}^{-1}$), *A* is the geometric electrode area (0.189 cm^2^), *n* is the number of electrons transferred in the electrochemical process (*n* = 1), *C* is the concentration of the redox probe (1 mM) and υ is the applied voltammetric scan rate (V s^–1^). The electronic supplementary material, figure S6 shows the excellent correlation between the theoretically predicted and experimentally observed response. The electrochemical responses of the EPPG and BPPG are in excellent agreement with previous literature [9,26] and are expected given the differing global coverage of edge plane like- sites/defects [1,11,12,24].

In order to electrically wire/connect to the GO, the most common approach is to modify existing well-known/characterized electrode materials and explore the response of GO modified surfaces; such an approach is commonly adopted in the literature and this is exactly what we wish to mimic. The voltammetric profiles of GO modified electrodes were next explored, with the responses shown in figure 1. It is evident that upon modification of the EPPG and BPPG electrode surfaces with increasing masses of GO, the voltammetric profiles are considerably altered. In the case of the BPPG electrode, the Δ*E*_P_ decreases from 183.1 (BPPG) to 78.1 mV after modification with up to 49.5 µg GO (at 100 mV s^–1^ versus SCE). On the other hand, when using an underlying substrate that exhibits fast HET kinetics, as is evident at the EPPG electrode, the initial introduction of 1.4 µg GO increases the relatively small Δ*E*_P_ of 68.4 (EPPG) to 100.1 mV (at 100 mV s^–1^ versus SCE), indicating a shift to a slower HET process; which then improves with further additions of GO and returns to a value superior to that originally obtained at the bare (unmodified) EPPG after modification with up to 49.5 µg GO (Δ*E*_P_ of 63.5 mV at 100 mV s^–1^ versus SCE). Potential changes in the mass transport regime, i.e. going from that of semi-infinite planar diffusion to that of a thin-layer response [44], were explored for the GO modified EPPG and BPPG electrodes in the form of scan rate studies. As shown later (*vide infra*) the response obtained is *not* that of thin-layer and thus given that the process remains under the control of semi-infinite planar diffusion, the improved/beneficial response (Δ*E*_P_) observed using the GO modified electrodes is probably because of to the material itself rather than mass-transport changes.

What is evident is that as the graphite electrodes are modified with increasing amounts of GO (*viz* figure 1) the magnitude of the voltammetric peak heights decrease. Since one is modifying an electrode surface with a new material, this is clearly resulting in a new electrode surface with a different morphology, a differing electrode area and different electrochemical activity than that of the original underlying surface. As such, as a favourable electrochemical response (i.e. smaller Δ*E*_P_ and increased reversibility) is obtained at GO modified electrodes, which is indicative of the GO giving rise to improvements in the electrochemical reversibility of the electrochemical process, suggesting that GO gives rise to increased electron transfer kinetics, which probably arise because of the degree of edge plane defects present on the GO material in addition to the process being mediated by the presence of favourable oxygenated species to an extent; it has been noted that oxygenated species can alter the observed electrochemistry of this redox probe [24].

**Figure 2.**
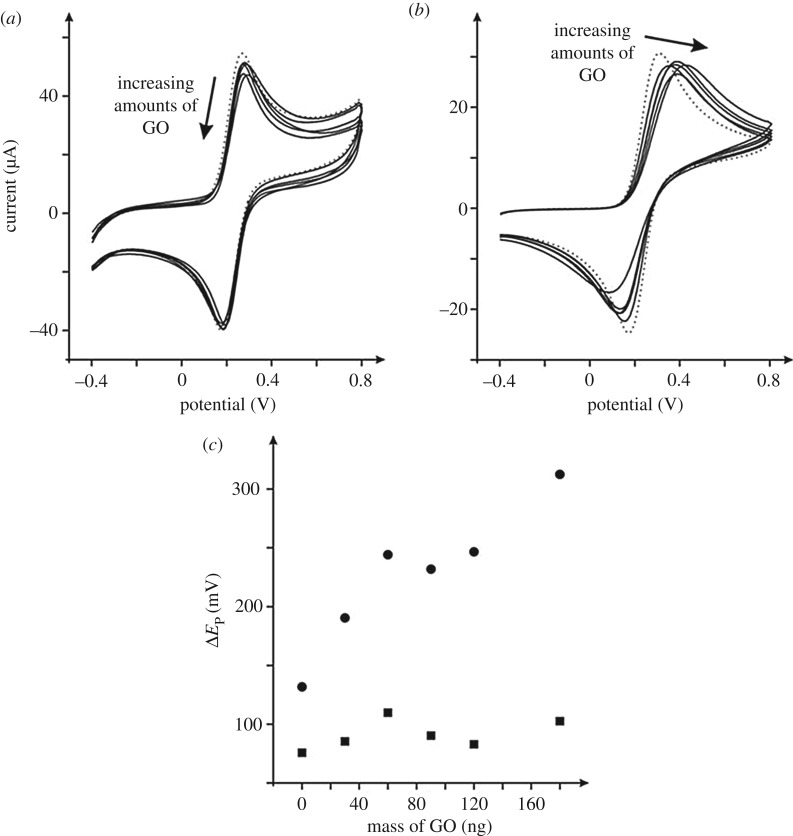
Cyclic voltammetric responses of ng amounts of GO modified EPPG (*a*), and BPPG (*b*) electrodes recorded in 1 mM ferrocyanide/0.1 M KCl. All data are obtained at a scan rate of 100 mV s^–1^ (versus SCE). The response of the unmodified/bare EPPG and BPPG is shown in (*a*) and (*b*) as the dotted lines respectively. (*c*) The analysis of the peak-to-peak separation (Δ*E*_P_) from (*a*) and (*b*) as a function of GO coverage; circles represent BPPG and squares the EPPG electrode respectively.

The effect of GO coverage, a critical parameter often overlooked in the literature, is next considered and as shown in figure 2, lower coverages than those shown in figure 1 are explored, which appear to ‘block’ the underlying electrode, that is, give rise to a worse response since the peak-to-peak separations become larger, indicating that the rate of electron transfer has reduced. Such an observation is reminiscent of the two behavioural zones defined in previous work when dealing with electrode substrates modified with pristine graphene [9], where at very low coverages a ‘zone I’ is encountered, resulting in a different response to that observed in ‘zone II’, which is at greater coverages, such that the electrochemical characteristics of the material under investigation are altered. Previous work exploring pristine graphene modified EPPG and BPPG electrodes demonstrated that the underlying/supporting electrode critically affects the orientation of the immobilized graphene, giving rise to differing voltammetric responses [9,10]. Indeed the same is true here for GO modified EPPG and BPPG surfaces.

The electrochemical response of other commonly used redox probes, hexaammine-ruthenium(III) chloride and hexachloroiridate(III) have been explored previously [36]. The oxygenated species present on the GO react chemically with the reduced and oxidized forms of ruthenium(III) and iridium(III) respectively in what is termed an *EC*′ reaction, where ultimately there is a regeneration of the starting reactant which then undergoes the electrochemical reaction again, this is what gives rise to a unique voltammetric response. Unfortunately, owing to said unique voltammetry it is not possible to characterize the electronic structure of the GO using these two probes, although this response is useful for other applications; for further information readers are referred to ref. [36] or the electronic supplementary material, figure S7 where the data have been reproduced.

**Figure 3.**
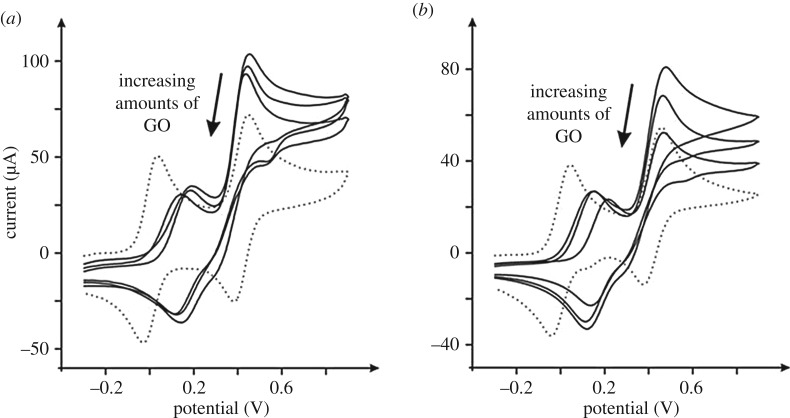
Cyclic voltammetric responses of 2.75, 5.50 and 11.0 µg GO modified EPPG (*a*), and BPPG (*b*) electrodes recorded in 1 mM TMPD (pH 7 PBS). All data are obtained at a scan rate of 100 mV s^–1^ (versus SCE). The response of the unmodified/bare EPPG and BPPG is shown in (*a*) and (*b*) as the dotted lines respectively.

The voltammetric response of N,N,N′,N′-tetramethyl-p-phenylenediamine (TMPD) is next sought at the GO modified electrodes. In aqueous solutions, as shown in figure 3, two *quasi*-reversible electrochemical processes are observed, with the first electrochemical oxidation peak at *ca* +0.04 V (EPPG versus SCE at 100 mV s^–1^) owing to the electrochemical process: $TMPD-e^{-}\to TMPD^{•+}$, and the second oxidation process, where a peak is observed at *ca* +0.42 V (EPPG versus SCE at 100 mV s^–1^), owing to the following process: $TMPD^{•+}-e^{-}\to TMPD^{2+}$. Interestingly, the electrochemical process of TMPD upon bare carbon electrodes is scan rate dependent, as shown in the electronic supplementary material, figure S8, and figure 3, where at ‘fast’ scan rates the voltammetric process is observed, but when using ‘slow’ scan rates the reduction wave at *ca* +0.29 V is significantly reduced. This is owing to a chemical process where water reacts with the electrochemically generated TMPD^2+^ species (which originates from the second oxidation process described above), such that on the reverse scan the TMPD^2+^ that would be seen electrochemically reduced is lost (since it has been depleted *via* a chemical reaction with water) and hence the voltammetric peak is reduced (thus whether or not a voltammetric reverse peak is observed will be owing to the respective rates of reaction, chemical versus electrochemical). In the case of GO modified electrodes, as shown in figure 3, a significant change in the voltammetric response is evident over that of the bare electrode. Note that this is quantitatively similar to the case reported above where the voltammetric response alters upon changing the scan rate (see the electronic supplementary material, figure S8) but in this case (figure 3) the scan rate is fixed and only the surface is changing through the introduction of GO. The GO results in a change in the reversibility of the first electrochemical process ($TMPD-e^{-}\to TMPD^{•+}$; note the shift in potential and reduction in peak height) and the second *quasi*-reversible reduction wave is significantly lost. Thus, this voltammetric response demonstrates that: (i) the addition of GO reduces the available edge plane sites needed for the electrochemical oxidation of the first electrochemical process ($TMPD-e^{-}\to TMPD^{•+}$); and (ii) the oxygenated groups on the GO accelerate the chemical process of TMPD^2+^ reacting with water (see above) or react themselves with TMPD^2+^ to form an new product, which is probably electrochemically inactive. Thus, it is clear that, by its inherent nature, GO can alter electrochemical mechanisms, which has not been seen for other graphene alternatives.

### 3.2. Graphene oxide evaluated towards the sensing of epinephrine

We now turn to exploring the electrochemical response towards epinephrine and the potential sensing capabilities of GO to see if this can give rise to improved electroanalytical performances. We use epinephrine (a catecholamine neurotransmitter) because it is an important analyte, playing a pivotal role in the mammalian central and sympathetic nervous and cardiovascular systems, where the concentration of this hormone within the blood is indicative of both physical and mental stress levels [45]. Epinephrine has also been extensively studied at a range of electrode materials, which allows us to benchmark the response of the GO.

**Figure 4.**
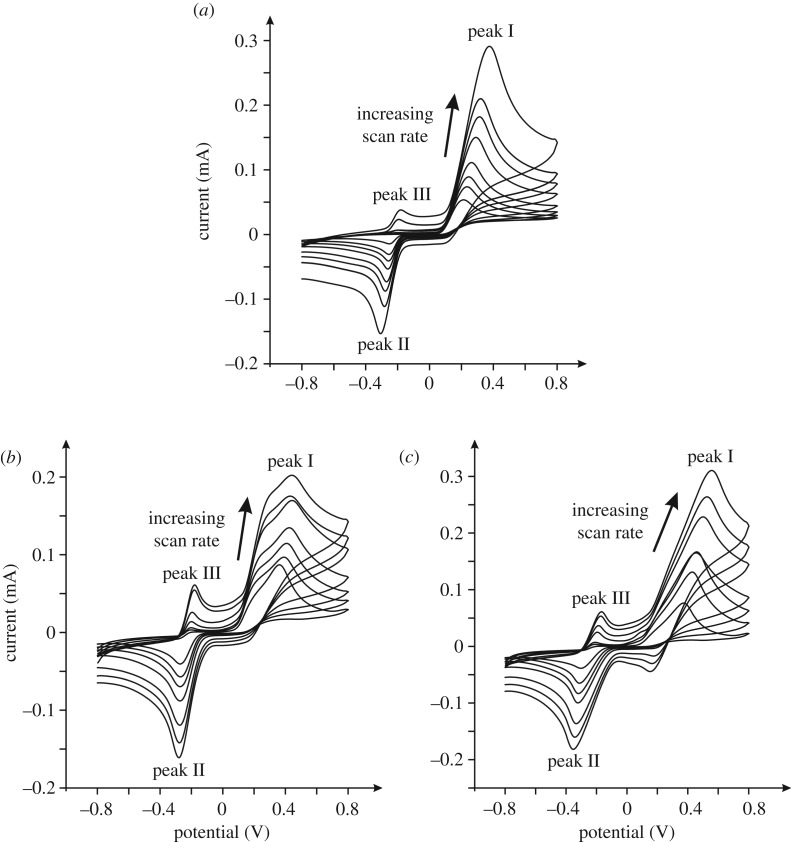
Cyclic voltammetric profiles recorded in 1 mM epinephrine/pH 7 PBS (versus SCE). (*a*) Responses of a bare/unmodified EPPG electrode recorded at a range of scan rates: 0.01–0.5 V s^–1^. (*b*) and (*c*) were recorded using a 16.5 µg GO modified EPPG (*b*), and BPPG (*c*) electrode respectively at a range of scan rates: 0.01–0.2 V s^–1^.

**Scheme 1.**
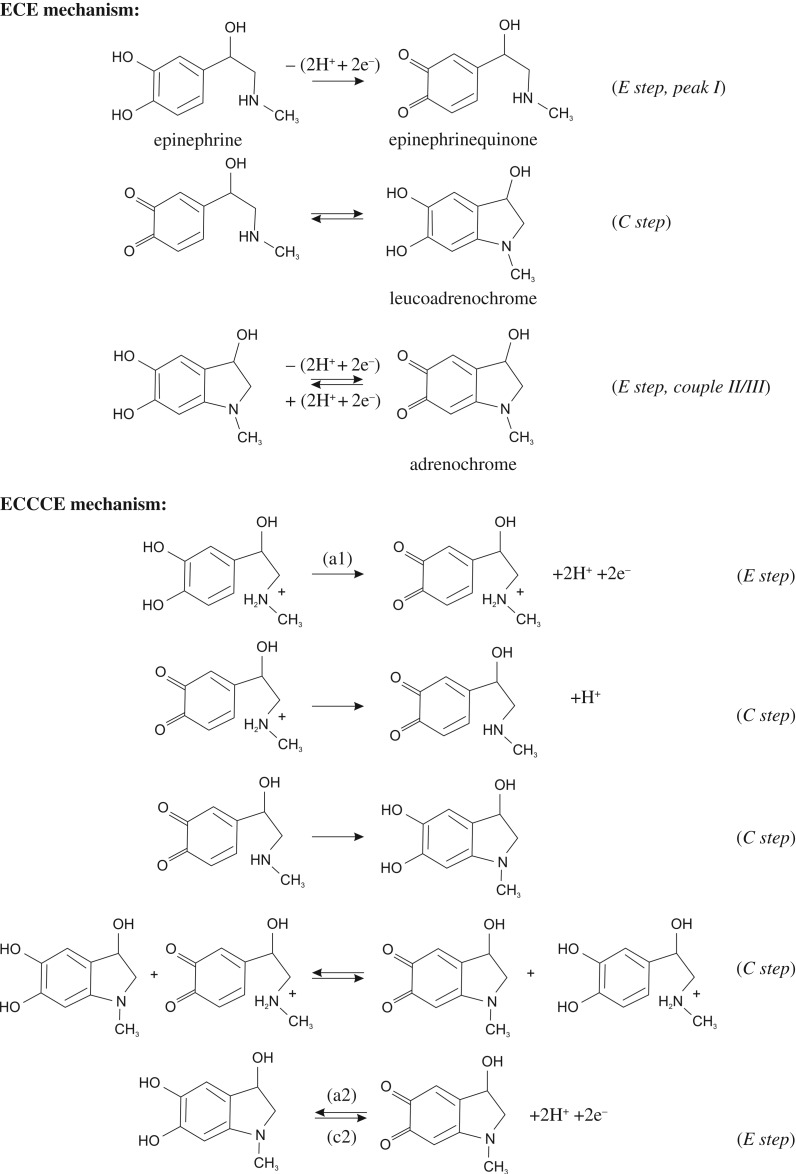
The electrochemical oxidation of epinephrine, which undergoes an electron transfer-chemical reaction-electron transfer (ECE) mechanism and a more detailed ECCCE mechanism. Adapted from refs [46–48].

The electrochemical behaviour of a bare/unmodified EPPG electrode was sought in a pH 7 phosphate buffer solution (PBS) containing 1 mM epinephrine with the voltammetric response explored over a range of scan rates (0.01–0.5 V s^–1^). Figure 4*a* depicts typical voltammetric profiles where a large voltammetric peak is observed *ca* +0.38 V (versus SCE, 0.5 V s^–1^), labelled peak I, with an additional two peaks at *ca* −0.30 V and −0.22 V (versus SCE, 0.5 V s^–1^) labelled as peak II and peak III (couple II/III) respectively. The voltammetric response of epinephrine is well-known and undergoes an electrochemical-chemical-electrochemical (ECE) reaction, that is, an electrochemical processes followed by a chemical and then a further electrochemical step [46,47], or can be described in more detail as an electrochemical-chemical-chemical-chemical-electrochemical (ECCCE) reaction [48]. The electrochemical mechanism is summarized in scheme 1. The electrochemical oxidation of epinephrine undergoes a two electron and two proton process to form epinephrinequinone (scheme 1, E step) giving rise to peak I (figure 4*a*).

**Figure 5.**
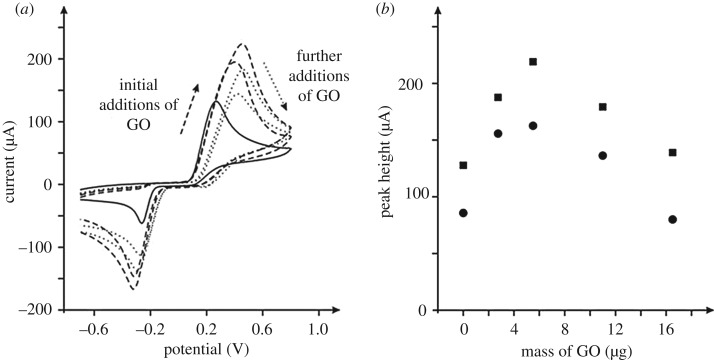
Typical cyclic voltammetric responses (*a*) of GO modified EPPG electrodes recorded in a 1 mM epinephrine (pH 7) PBS. The response of an unmodified/bare EPPG electrode is also shown (solid line). Scan rate: 100 mV s^–1^ (versus SCE). (*b*) The analysis (using peak I (see figure 4*a*)) of the voltammetric peak height from (*a*) as a function of GO additions; squares represent the EPPG and circles the BPPG electrode respectively.

This electrochemically formed product undergoes a chemical step (scheme 1, C step) *via* a ring closure process forming leucoadrenochrome. This is electrochemically active and is more easily oxidized and has its own redox couple (scheme 1, E step), giving rise to the corresponding voltammetric peaks labelled as II/III in figure 4*a*. Note that in this experiment (figure 4*a*) the voltammetric response was run from slow to fast scan rates with the solution agitated in-between scan rates, and in doing so the ECE process occurs and appreciable amounts of adrenochrome (scheme 1) are formed such that at the faster and consequently later scan rates, the redox couple corresponding to peaks II/III in figure 4*a* are observed prior to the first initial E step (i.e. epinephrine to epinephrinequionone). Figures 4*b*,*c* and 5 show the typical response towards epinephrine from using GO modified electrodes. It is interesting to observe that the redox couple II/III (figure 4*b*,*c*) is more pronounced and that the voltammetric peak height of the main epinephrine oxidation signal (figure 5, peak I) is greater in magnitude than that observed at the bare graphite electrodes. A scan rate study was explored using the GO modified electrodes, with the main epinephrine signal explored as a function of scan rate (figure 4*b*,*c*, peak I). The peak position was observed to shift with scan rate and the magnitude of the signal increased. Analysis of the peak height as a function of scan rate and square-root of scan rate revealed a highly linear response in terms of the former, indicating that the electrochemical mechanism at the GO modified electrodes operate *via* a diffusional process. Furthermore, this was observed to be the case when monitoring the other redox couple present also, with the response again dependent on scan rate and thus no thin-layer effects are evident, meaning that the electrochemical responses are under diffusional influence in each case. Where nanomaterials are used, thin-layer effects can result where a porous surface is produced, which can change the mass transport regime from planar/linear to that of a thin-layer type cell/response, which changes the voltammetry and can be misinterpreted as the nanomaterial being electrocatalytic [44,49]. Again, to reiterate, from inspection of our voltammetric responses (data presented above) and indeed comparison of our voltammetry with single-walled carbon nanotube (SWCNT) modified glassy carbon (GC) electrodes towards the detection of epinephrine where the electrochemical oxidation wave dramatically (in this case a lack of diffusional tail is evident) changed compared to a bare/unmodified GC [50], it is concluded that there are no thin-layer effects evident/present.

The effect of pH upon the voltammetric response (peak I) using the GO modified electrodes was explored over the pH range of 1.04 to 8.1 (p*K*a* *= 8.88) [46]. A plot of peak potential versus pH indicated that the peak potential moves to more negative values with an increase in pH; linear regression gave slopes of 0.066 V per pH unit (data not shown), indicating that the transfer of electrons is accompanied by an equal number of protons in the electrochemical oxidation mechanism of epinephrine to epinephrinequinone (scheme 1), which is in good agreement with literature using a range of electrode substrates [46,51,52].

The effect of changing the GO coverage/amount was also explored. Figure 5 shows the voltammetric response of changing the GO coverage where it can be seen that the electrochemical response is coverage dependent and that an initial response is observed where the voltammetric peak height increases up to a maxima, after which the response then decreases upon further mass-additions of GO. That is to say that, initially a beneficial response is observed, beyond which further additions of GO become detrimental.

**Figure 6.**
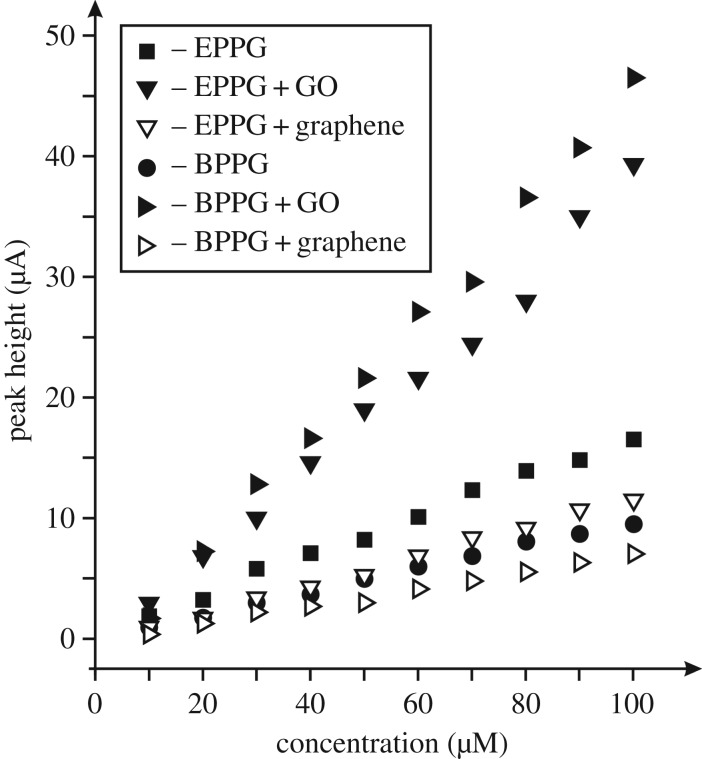
Typical calibration plots resulting from the analysis of cyclic voltammograms from increasing additions of epinephrine made into a pH 7 PBS using a range of electrodes. All data obtained at a scan rate of 100 mV s^–1^ (versus SCE). GO modified electrodes used 5.5 µg and graphene modification were with 20 ng of the respective materials.

Next the electroanalytical response of the GO modified electrodes towards the sensing of epinephrine was evaluated. Figure 6 shows the resulting calibration plots, which compares the response of not only GO but pristine graphene. It is evident from inspection of figure 6 that the optimal response is observed for the case of the GO modified BPPG, which is greatly enhanced. The origin of the improved response towards the sensing of epinephrine is likely to arise from the C/O functionalities, in particular, energetically favourable hydrogen bonding between the OH groups on the epinephrine and the COOH moieties on the GO. Insights from the literature using a penicillamine (Pen) self-assembled monolayer modified gold electrode towards the sensing of epinephrine reported an enhancement in the sensing over a bare/unmodified gold electrode owing to hydrogen bonding between the COOH on the Pen and OH groups on the epinephrine [53]; such independent work adds weight to our hypothesis. As observed in figure 5, the voltammetric response becomes detrimental at higher coverages, where it is likely that the increased proportion of oxygen groups repel the target analyte. One might consider that the electrochemical reaction operating at the GO modified electrode is altered owing to the large amount of C/O moieties. In fact, with the introduction of GO we see a greater increase in all of the voltammetric peaks and hence a better electrochemical surface has resulted; a larger amount of product is electrochemically oxidized in the first E step (scheme 1) as is evident from the larger voltammetric peak currents, which results in more product undergoing the C step and is then available for the corresponding last E step—hence the couple II/III is enhanced. Insight from the literature has shown similar responses for a platinum bare/unmodified electrode explored towards the sensing of epinephrine [48].

Next, the electroanalytical response of the GO modified electrodes towards the sensing of epinephrine was evaluated using the optimal coverage as determined from figure 5. Figure 6 shows the subsequent calibration plots resulting from bare/unmodified EPPG and BPPG electrodes, exhibiting analytical sensitivities of 0.16 and 0.09 A M^–1^ respectively, which substantially increase to 0.39 and 0.49 A M^–1^ respectively when modified with 5.5 µg GO. As a control experiment, pristine graphene was explored, where from inspection of figure 6 it can be clearly observed that the introduction of graphene results in reduced analytical sensitivities in both instances, owing to the pristine nature of the graphene (no oxygen species) and its low density of edge plane- like/sites [9–12,15,54,55]. The unfavourable analytical sensitivities resulting from decreased peak heights at 20 ng graphene modified EPPG and BPPG electrodes were 0.11 and 0.07 A M^–1^ respectively. Clearly, GO can provide enhancements in the voltammetric peak current and towards analytical sensitivities, which have potential to be electro-analytically exploited.

### 3.3. Graphene oxide evaluated towards the sensing of dopamine

**Figure 7.**
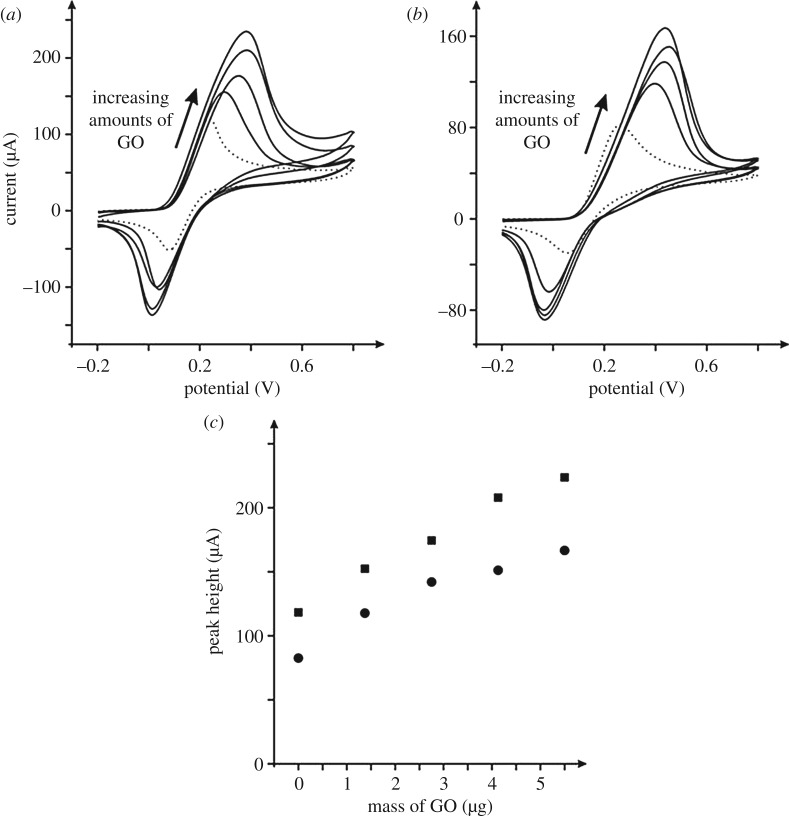
Typical cyclic voltammetric responses (*a*) of GO modified EPPG electrodes, and (*b*) GO modified BPPG electrodes recorded in a 1 mM dopamine (pH 7) PBS. The response of an unmodified/bare EPPG and BPPG electrodes are shown (dotted lines) in (*a*) and (*b*) respectively. Scan rate: 100 mV s^–1^ (versus SCE). (*c*) The analysis of the voltammetric peak height from (*a*) and (*b*) as a function of GO additions; squares represent the EPPG and circles the BPPG electrode respectively.

The electrochemical response of dopamine was considered as it is of great significance given that it is an important neurotransmitter that plays a pivotal role in the function of the hormonal, renal and central nervous systems [56]. Figure 7 shows the voltammetric response of bare/unmodified EPPG and BPPG electrodes, the responses of which are consistent with the literature [57]. The introduction of GO onto the electrode surface results in a large increment in the voltammetric current, which increases in a linear fashion. The effect of changing the voltammetric scan rate upon the peak current was explored with a plot of peak height versus square-root of scan rate found to be linear, indicating a diffusional electrochemical process and the absence of any thin-layer effects.

The effect of pH upon the electrochemical detection of dopamine when using the GO modified electrodes was explored over the pH range of 1.04 to 8.1 (p*K*a* *= 8.92) [46]. A plot of peak potential versus pH indicates that the peak potential moves to more negative values with an increase in pH. The linear regression of this plot gave a slope of 0.061 V per pH unit (data not shown), indicating that the electrochemical oxidation of dopamine involves an equal number of electrons and protons, likely to be 2. In the electrochemical oxidation mechanism, dopamine is electrochemically oxidized to an open-chain quinone, dopamine quinone, where the reduction wave corresponds to the open-chain quinone (dopamine quinone) being reduced to dopamine. The beneficial response of the GO is similar to that described above with epinephrine in that it is well known that dopamine needs to adsorb upon the electrode surface [2,58] and that the large amount of oxygen species enhances this process and/or along with hydrogen bonding (*vide supra*).

**Figure 8.**
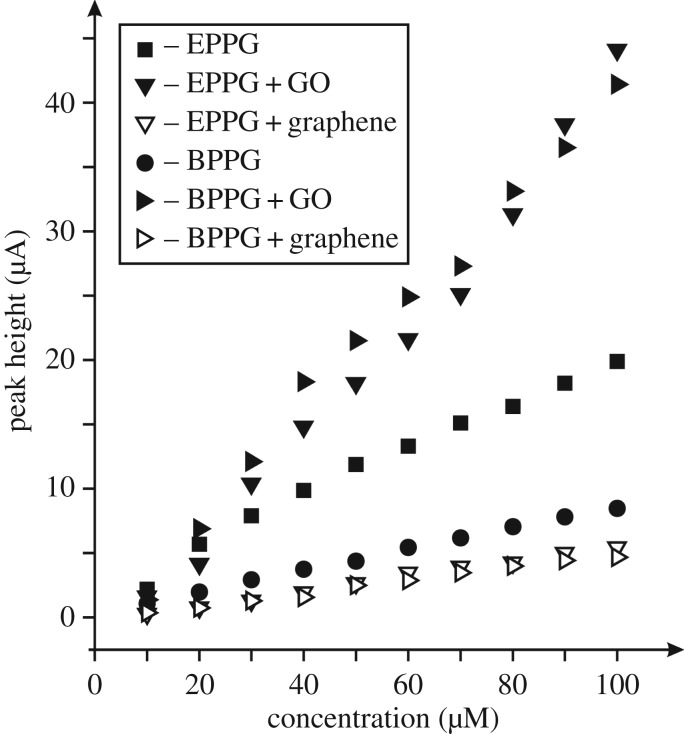
Typical calibration plots resulting from the analysis of cyclic voltammograms from increasing additions of dopamine made into a pH 7 PBS using a range of electrodes. All data obtained at a scan rate of 100 mV s^–1^ (versus SCE). GO modified electrodes used 5.5 µg and graphene modification were with 20 ng of the respective materials.

Last, the electroanalytical response of the GO modified electrodes towards the sensing of dopamine was appraised. Figure 8 shows the resultant calibration plots from using bare/unmodified EPPG and BPPG electrodes and following modification with GO (and for comparison, graphene). It is clear that bare BPPG and EPPG electrodes exhibit analytical sensitivities of 0.08 and 0.19 A M^–1^ respectively, which is as expected for electrodes with differing global coverages of edge plane sites/defects [1,43]. The response of modifying the electrode with 5.5 µg GO clearly provides the most beneficial response with GO/EPPG and GO/BPPG giving improved analytical sensitivities of 0.46 and 0.43 A M^–1^ respectively. As a control, the response of 20 ng pristine graphene modified EPPG and BPPG electrodes are shown (0.06 and 0.05 A M^–1^, respectively) and do not provide any improvements, which is in agreement with prior literature [9,10,12,54]. The electrochemistry of dopamine is known to be surface sensitive requiring adsorption sites [2,58] and the use of GO with its numerous C/O moieties enhances the electrochemical response as a result.

In summary, we have shown that for both dopamine and epinephrine, GO modified electrodes give superior electrochemical responses in terms of the electrochemical sensing abilities over that of the more traditional graphite and graphene based alternatives. It is important to emphasize that the response is coverage dependent and researchers are encouraged to undertake such control experiments to ensure they fully understand their electrochemical system/response; the current approach is to use one coverage and this might fall into a region that gives a detrimental response (i.e. see figure 5 which clearly shows a coverage dependency) and is consequently *falsely* attributed to be a poor electrode material. If the work presented herein is related to prior literature on graphene, which has been shown to give rise to substantial benefits, we suggest that GO is not reduced to graphene, but rather that GO itself can be beneficially used in a range of electrochemical applications.

## 4. Conclusion

The electrochemistry of GO has not been widely studied and in fact GO is used mainly as precursor to fabricating graphene. This is probably owing to the literature reporting that graphene is an insulator [38]. However, we have shown that the electrochemistry of GO gives rise to beneficial responses towards a range of analytes, which, critically, is coverage dependent. The electrochemical responses of GO towards the target analytes deviates from expected behaviour owing to the high amount of C/O moieties which dominate the voltammetric response. We have shown that these can be beneficially used for the sensing of electroactive biological analytes, with the response of GO superior to traditional graphite and graphene modified electrodes. It is important to identify that the voltammetric response of GO is coverage dependent, which is a contribution from the beneficial edge plane sites/defects upon the graphene surface and its edges and also the high proportion and diversity of oxygenated species present. Clearly, there are many fabrication routes for producing GO and each will make different varieties, this in turn will change the proportion and composition of edge sites and oxygenated species and will of course give rise to differing voltammetry; this makes the study of GO electrochemistry a fascinating subject.

This work proposes that *GO itself* has significant electrochemical and electrocatalytic properties with wide ranging potential for beneficial implementation into applications rather than simply being a precursor to fabricate graphene.

## Supplementary Material

Supplementary Information File
