# Supplementary material for: Graphene oxide electrochemistry: the electrochemistry of graphene oxide modified electrodes reveals coverage dependent beneficial electrocatalysis
Source: R Soc Open Sci. 2017 Nov 15;4(11):171128. doi: 10.1098/rsos.171128 (PMC5717673; doi:10.1098/rsos.171128)
Supplement: Supplementary Information File [file rsos171128supp1.docx]

**Electronic Supporting Information (ESI)**

**Graphene Oxide Electrochemistry: The Electrochemistry of Graphene Oxide Modified Electrodes Reveals Coverage Dependant Beneficial Electrocatalysis**

Dale A. C. Brownson^1^, Graham C. Smith^2^ and Craig E. Banks^1^*

*^1^: Faculty of Science and Engineering, Manchester Metropolitan University, Chester Street, Manchester, M1 5GD, UK.*

*^2^: Faculty of Science and Engineering, Department of Natural Sciences, University of Chester, Thornton Science Park, Pool Lane, Ince, Chester, CH2 4NU, UK.*

*To whom correspondence should be addressed.

Email: [c.banks@mmu.ac.uk](mailto:c.banks@mmu.ac.uk); Tel: ++(0)1612471196; Fax: ++(0)1612476831

Website: [www.craigbanksresearch.com](http://www.craigbanksresearch.com)

**Experimental details for the physicochemical characterisation**

Unless otherwise stated, the below experimental conditions/equipment was utilised to perform the characterisation reported on the materials studied herein. Transmission Electron Microscopy (TEM) images were obtained using a JEOL JEM-2010 (Oxford, Inca Energy TEM 100) using a 200 kV primary beam under conventional bright-field conditions. The sample was dispersed onto a holey-carbon film supported on a 300 mesh Cu TEM grid. Raman Spectroscopy was performed using a ‘Renishaw InVia’ spectrometer equipped with a confocal microscope (×50 objective) and an argon laser (514.3 nm excitation). Measurements were performed at a very low laser power level (0.8 mW) to avoid any heating effects. X-ray Diffraction (XRD) was performed using an “X'pert powder PANalytical” model with a copper source of Kα radiation (of 1.54 Å) and Kβ radiation (of 1.39 Å), using a thin sheet of nickel with an absorption edge of 1.49 Å to absorb Kβ radiation. A reflection transmission spinner stage (15 rpm) was implemented to hold the commercially sourced GO sample. To ensure well-defined peaks an exposure of 50 seconds per 2θ step was implemented with a size of 0.013°. The X-ray Photoelectron Spectroscopy (XPS) data was acquired using a bespoke ultra-high vacuum system fitted with a Specs GmbH Focus 500 monochromated Al Kα X-ray source, Specs GmbH Phoibos 150 mm mean radius hemispherical analyser with 9-channeltron detection, and a Specs GmbH FG20 charge neutralising electron
gun ^1^. Survey spectra were acquired over the binding energy range 1100–0 eV using a pass energy of 50 eV and high-resolution scans were made over the C 1s and O 1s lines using a pass energy of 20 eV. Under these conditions the full width at half maximum of the Ag 3d5/2 reference line is *ca*. 0.7 eV. In each case, the analysis was an area-average over a region approximately 1.4 mm in diameter on the sample surface, using the 7 mm diameter aperture and lens magnification of ×5. The energy scale of the instrument is calibrated according to ISO 15472, and the intensity scale is calibrated using an in-house method traceable to the UK National Physical Laboratory ^2^. Data were quantified using Scofield cross sections corrected for the energy dependencies of the electron attenuation lengths and the instrument transmission ^3^. Data interpretation was carried out using CasaXPS software v2.3.16 ^4^.

***Graphene Oxide (GO)* – Experimental details and physicochemical characterisation**

Commercially available GO was purchased from ‘Graphene Supermarket’ (Reading, MA, USA) ^5^ and consists of ‘single layered graphene oxide dispersed in water’ at a concentration of 275 mg L^–1^. The GO was synthesised using a modified Hummers oxidation method, that has been reported and characterised previously ^6, 7^, and produces graphene oxide (GO) which has an average flake size of between 0.5 and 5.0 micrometres and a thickness of 1 atomic layer; with at least 80% of the sample being single layer GO ^5, 8^. Additionally, the GO has been “base” washed in order to remove any oxidation debris.

Independent Raman spectroscopy, TEM, XPS and XRD analysis were all conducted.
ESI Figure 1A and 1B display TEM images of the GO platelets and indicates that they have an average particle size (lateral width) of *ca*. 2.0 µm which strongly agrees with size stated by the commercial manufacturer ^5^. Raman spectroscopy was utilised to confirm the presence of GO by structural characterisation, the obtained spectra is presented in ESI Figure 1C and displays the typical D and G vibrational band/peaks at *ca*. 1350 and 1590 cm^–1^ respectively. These characteristics are as expected and in agreement with the literature regarding GO ^9, 10^.
A combination of the D and G peaks gives rise to a 3S peak at 2910 cm^–1^ as a result of lattice disorders, as shown for graphene oxides, and furthermore, a characteristic wave is present at
*ca*. 2800 cm^–1^ corresponding to the 2D region ^7^. Additionally, the composition of the GO sample is confirmed *via* XRD (ESI Figure 1D) in which a characteristic ‘sharp’ peak is evident at
2θ = 11.5°, corresponding to the (001) diffraction peak of disordered GO ^11^. Last, XPS analysis was performed to determine the GO’s elemental composition, with ESI Figure 2 showing the gathered survey spectra and the individual spectra for the C and O regions. The GO was observed to contain 66.8 % atomic carbon and 28.6 % atomic oxygen with trace amounts of nitrogen, sulphur and chlorine, which are negligible contaminants present from the carrier solution/matrix. Specifically, groups corresponding to graphitic C–C bonding in addition to C–O or C–O–C bonds (47.21 %, 286.7 eV) and C=O or COO (7.94 %, 288.4 eV) bonds where characteristically present, which is in excellent agreement with previous literature reports regarding GO ^9, 10^.

The combination of surface and physicochemical analysis presented above confirms that the commercially sourced GO utilised herein is of a high quality/purity.

***Pristine* *graphene* – Experimental details and physicochemical characterisation**

Pristine graphene was commercially obtained from ‘Graphene Supermarket’
(Reading, MA, USA) ^5^ and is reported to be produced *via* a substrate-free gas-phase synthesis method ^5, 12-14^. This single-step technique involves sending an aerosol consisting of liquid ethanol droplets and argon gas directly into a microwave-generated argon plasma
(at atmospheric-pressure), where over a time scale in the order of 10^–1^ s, ethanol droplets evaporate and dissociate in the plasma forming solid matter that through characterisation by TEM and Raman spectroscopy is confirmed to be true graphene ^12, 13^. The fabricated graphene sheets are sonicated in ethanol to form a homogeneous suspension before being distributed by the supplier ^5, 14^.

Independent TEM and Raman analysis of the commercially sourced graphene (as received from the supplier and consequently as used throughout this work) is presented in ESI Figure 3 and ESI Figure 4 respectively. ESI Figure 3 depicts typical TEM images of the commercially sourced graphene. It is evident that the graphene domains comprise of predominantly of single-layer graphene sheets, which appear to exhibit an intraplanar microcrystalline size, *L_a_* of between 500 and 5000 nm and an average interplanar microcrystalline size, *L_c_* of *ca*. 0.34 nm (one monolayer), which compares well to pristine graphene as reported theoretically in the literature ^15^.
ESI Figure 4 depicts the Raman spectrum of the commercially sourced graphene. The Raman spectrum reveals two characteristic peaks at *ca*. 1580 and 2680 cm^–1^, which are due to the G and 2D (G’) bands respectively. The highly symmetrical 2D (G’) peak indicates that the surface is comprised of single-layer graphene (consistent with TEM images) ^16^. Additionally, the intensity ratio of the G and 2D bands (G/2D = 0.61) indicates that the graphene sheets are indeed comprised of single-layer graphene domains, where the low intensity of the G band in relation to the 2D peak is characteristic of monolayer graphene ^16^. The presence of a small D band (1330 cm^–1^) indicates a small number of structural defects on the graphene surface (limited basal plane crystal defects), however, the relatively low intensity of the D band, which is not easily distinguishable from the ‘base line’, suggests that an ordered graphene structure is present which is of high quality and thus represents that of pristine graphene in nature ^15, 16^. XPS chemical analyses revealed the material to comprise of 95.84 % atomic carbon and 4.16 % atomic oxygen; the low O/C ratio suggests pristine graphene ^15, 16^.

Through detailed inspection of the above presented independent analysis (TEM, Raman spectroscopy and XPS), one can clearly confirm the presence of single layered ‘pristine’ graphene sheets that possess low oxygen content and a low coverage of edge plane like-site/defects.

Note, control experiments were performed in terms of ethanol modified electrodes for the purpose of de-convoluting the origin of the electro-activity and ensuring that electrochemical responses observed were not a result of the solvents utilised; such control experiments revealed that ethanol has no effect upon electro-activity.

**ESI Figure S1**

Characterisation of the commercially sourced GO; (A) and (B) TEM images of GO nanosheets (Scale bar: 500 and 100 nm respectively), (C) Raman spectra of GO deposited onto a silicon wafer and (D) XRD spectra.


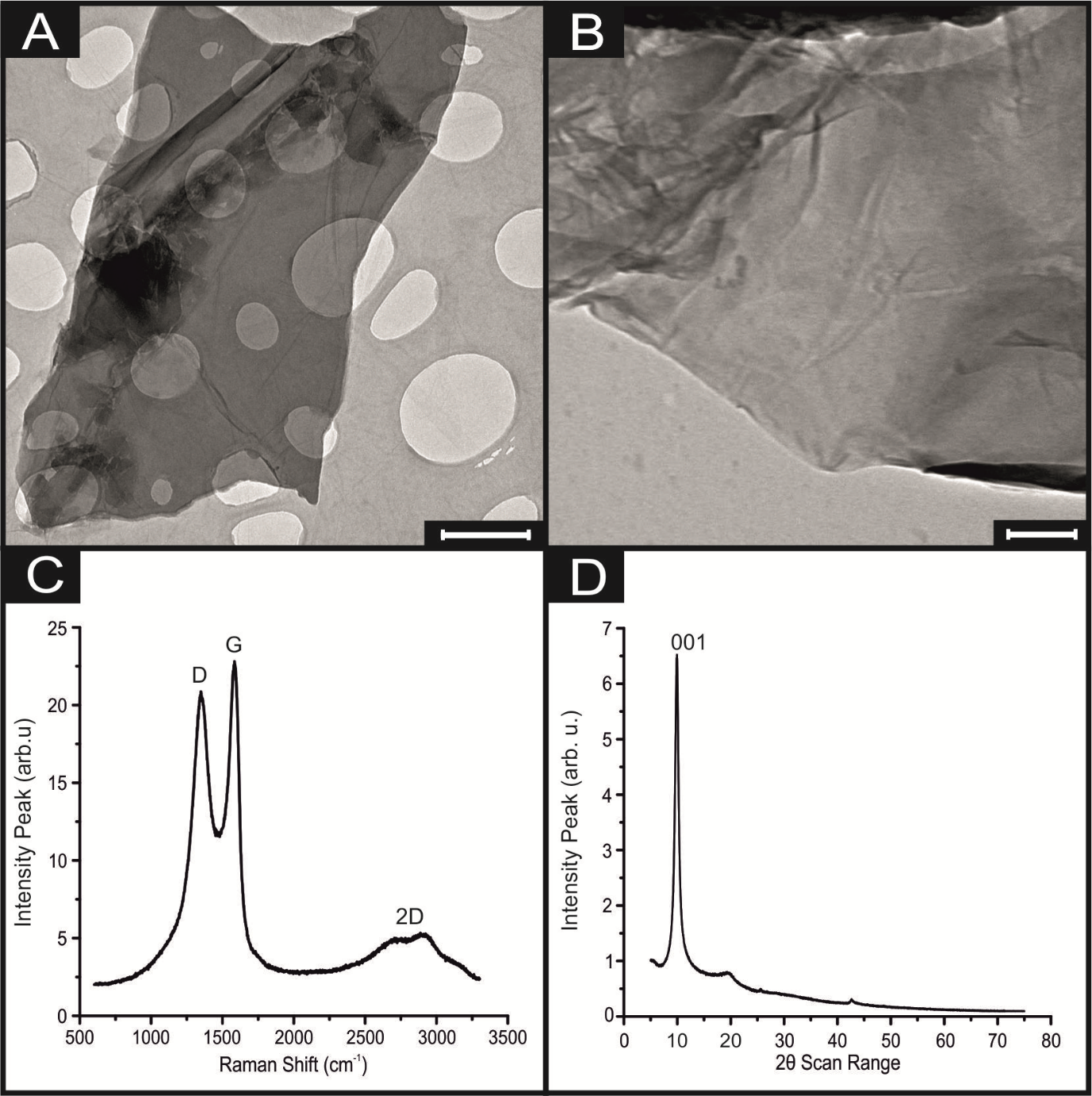


**ESI Figure S2**

High-resolution XPS spectra of C and O regions of the GO utilised herein (A and B respectively), with the full survey spectra also shown (C).


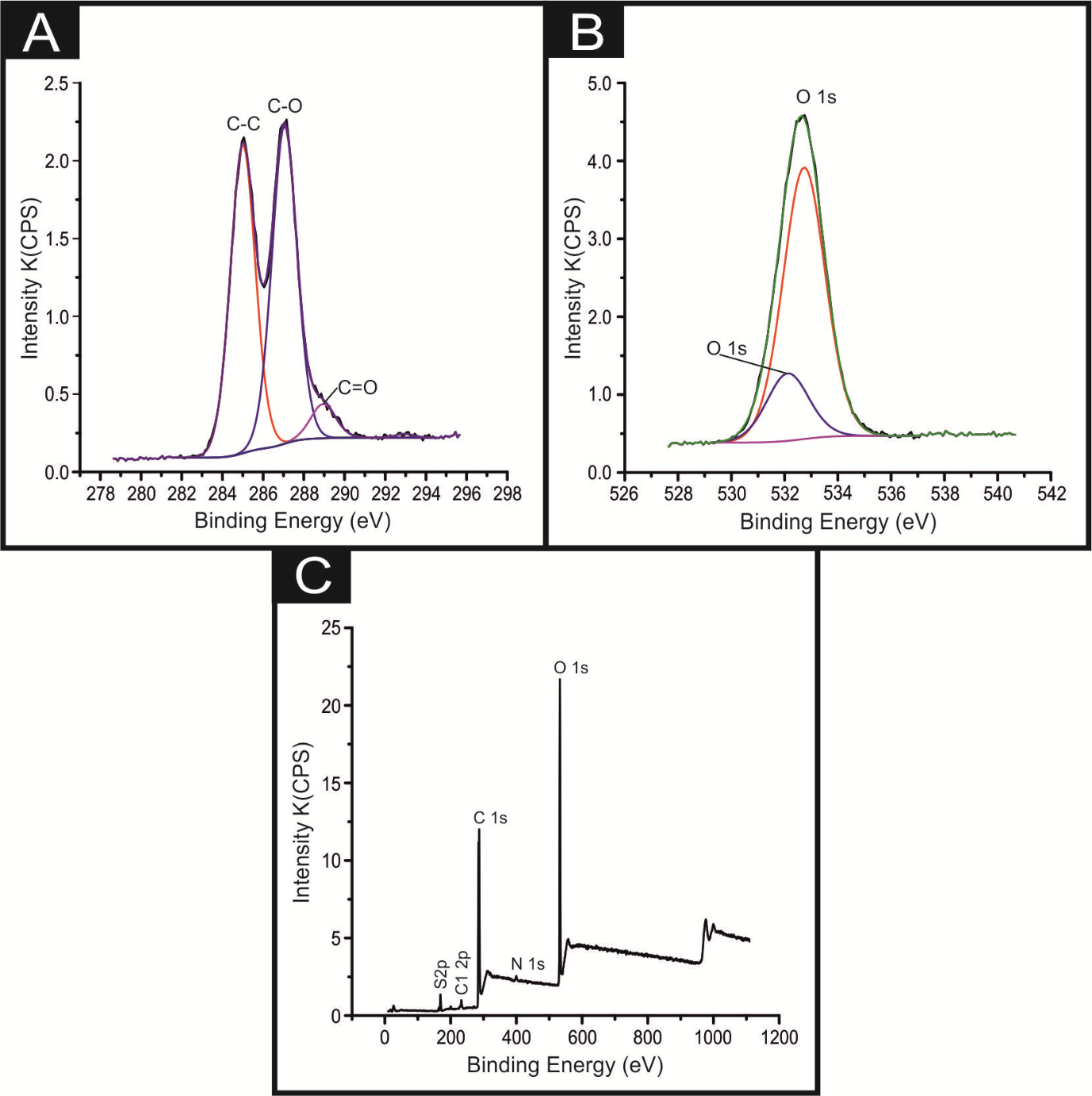


**ESI Figure S3**

Typical TEM images of the commercially obtained graphene sheets utilised in this work, images taken at increasing magnification.


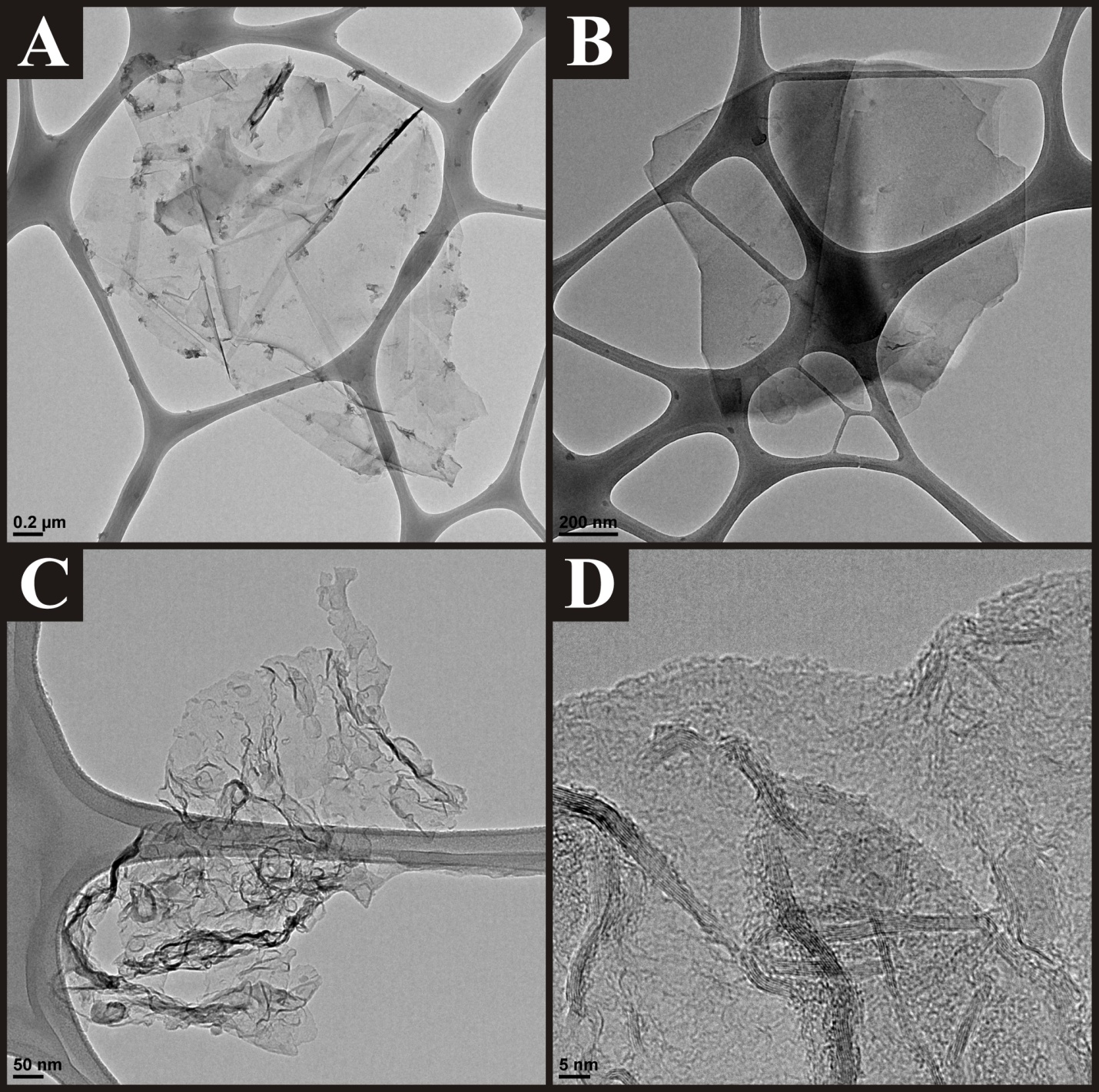


**ESI Figure S4**

A typical Raman spectrum of a commercially obtained graphene sheet as utilised in this study. Raman spectroscopy was performed after the graphene solution was deposited (and solvent allowed to evaporate) onto a quartz slide Si/SiO_2_ substrate (with a thickness of 300 nm SiO_2_ on Si). Raman spectra were recorded using LabRam (Jobin-Ivon) with a confocal microscope (100× objective) spectrometer with a He–Ne laser at 632 nm excitation at a very low laser power level (0.9 mW) to avoid any heating effect.


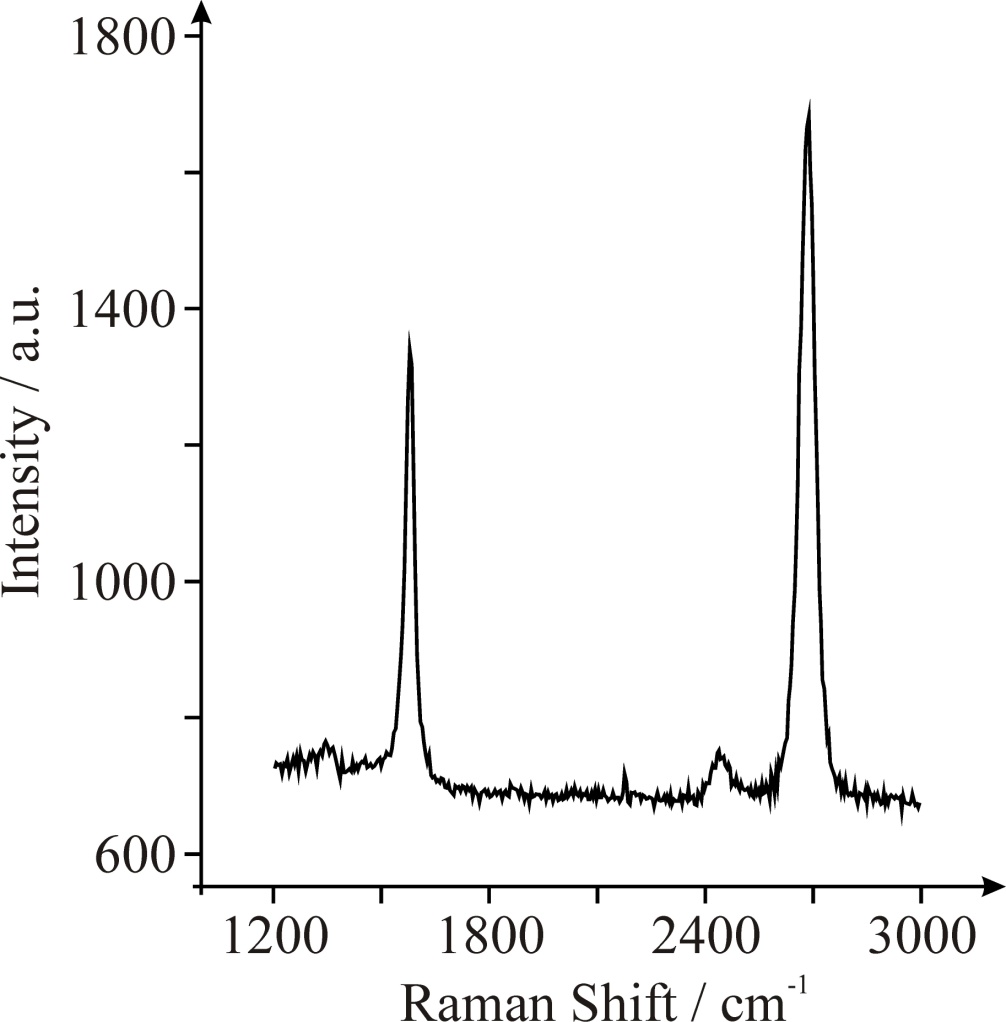


**ESI Figure S5**

Cyclic voltammetric profiles recorded in a pH 7 phosphate buffer solution (PBS) using unmodified EPPG (solid line) and 5.5 µg GO modified EPPG (dashed line) electrodes, where within both the anodic (A) and cathodic (B) potential regions there are no evident voltammetric peaks prior to the addition of our analytes and/or redox probes. Scan rate: 100 mVs^–1^ (*vs*. SCE).


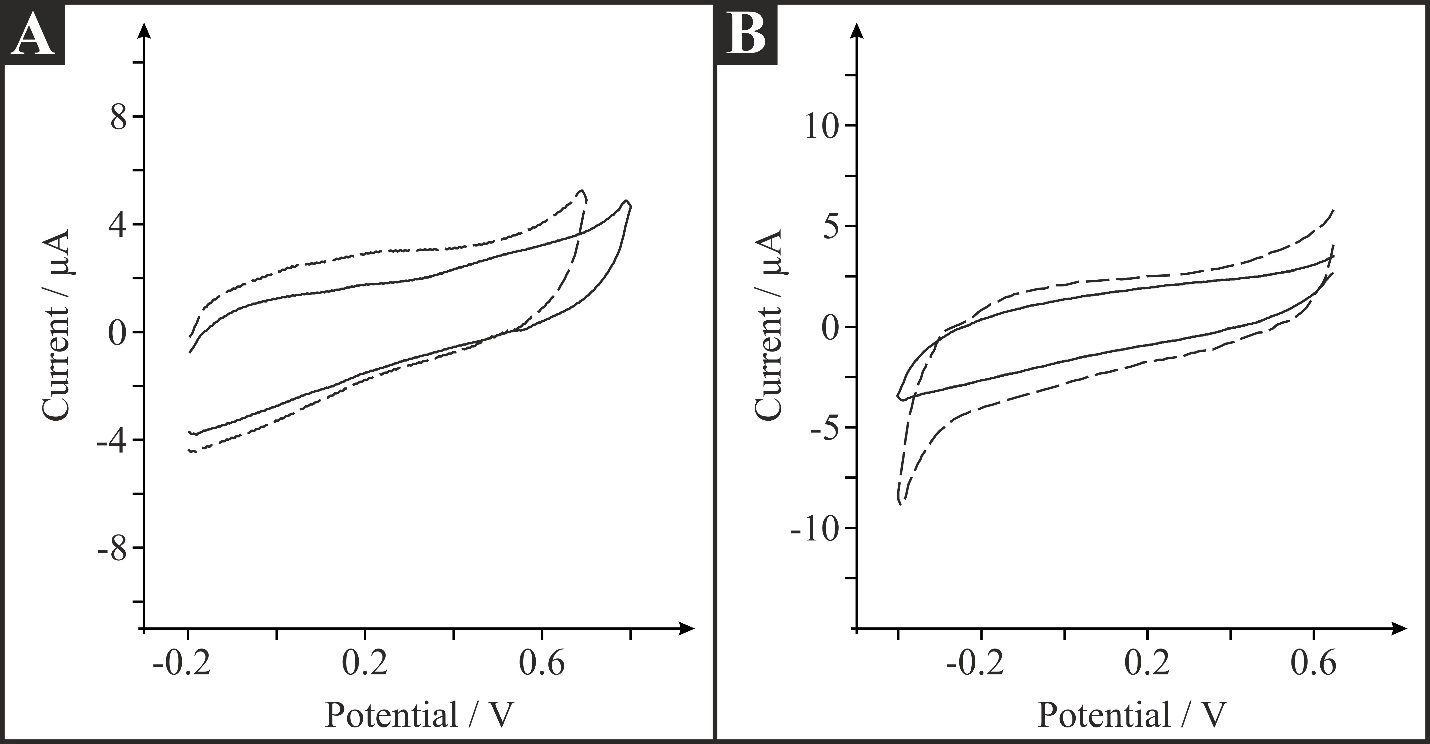


**ESI Figure S6**

Analysis of the observed peak currents as a function of the square-root of scan rate (squares). Also shown is the theoretically expected response (circles) using the Randles-Ševćik equation for a *quasi*-reversible electrochemical process; see main text for more details. Voltammetric probe:
1 mM potassium ferrocyanide(II) / 0.1 M KCl; Scan rate range: 0.005–1 Vs^–1.^ Electrode substrate: EPPG; T = 298 K.


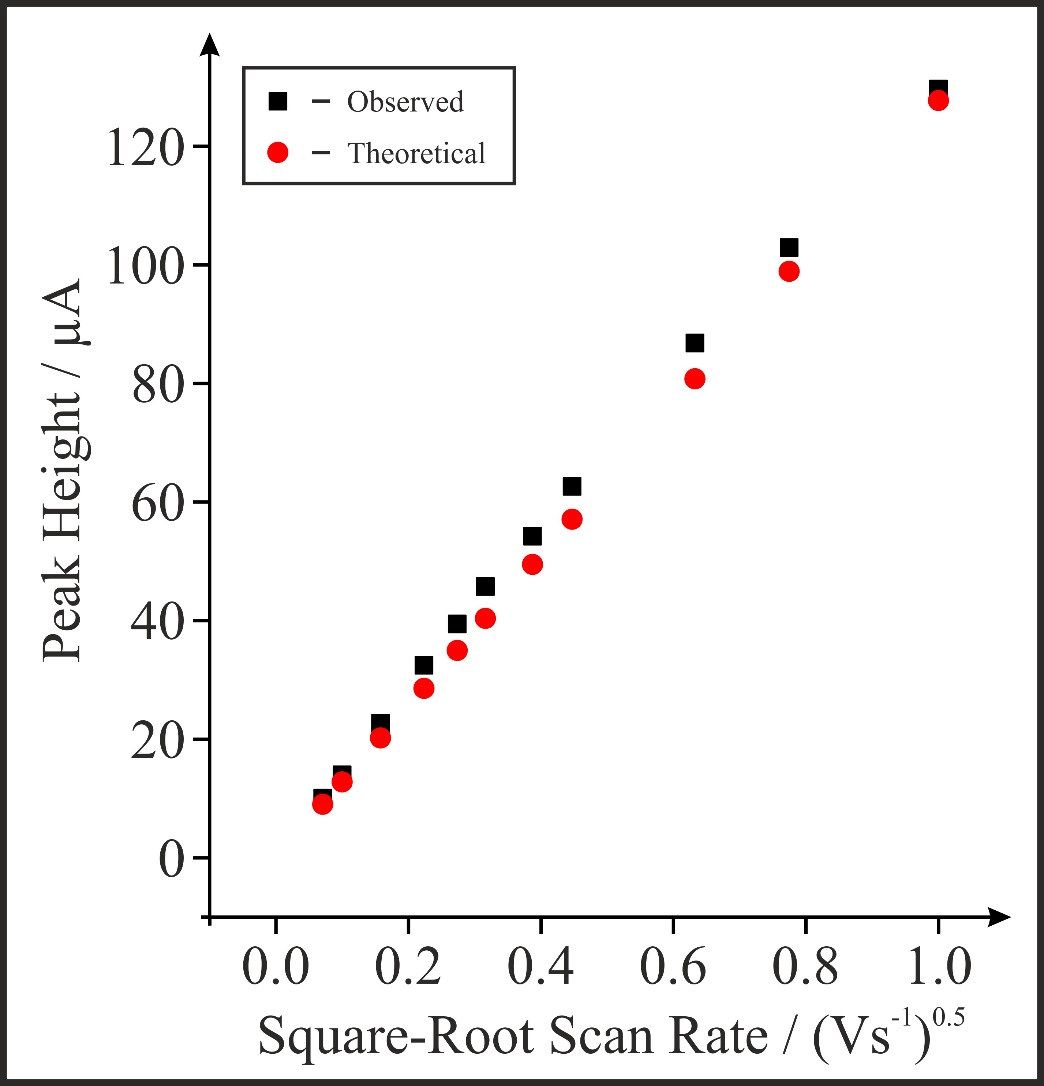


**ESI Figure S7**

Cyclic voltammetric profiles recorded towards (A) 1 mM hexaammine-ruthenium(III) chloride
/ 1 M KCl and (B) 1 mM potassium hexachloroiridate (III) / 1 M KCl. Responses were obtained using an EPPG electrode (dotted line) after modification with increasing depositions of (in A) 1.38, 2.75 and 8.25 μg GO (solid lines) and (in B) 1.38, 2.75 and 5.50 μg GO (solid lines).
Scan rate: 100 mVs^−1^ (*vs*. SCE). Adapted from Ref. [^8^] with permission of The Royal Society
of Chemistry.


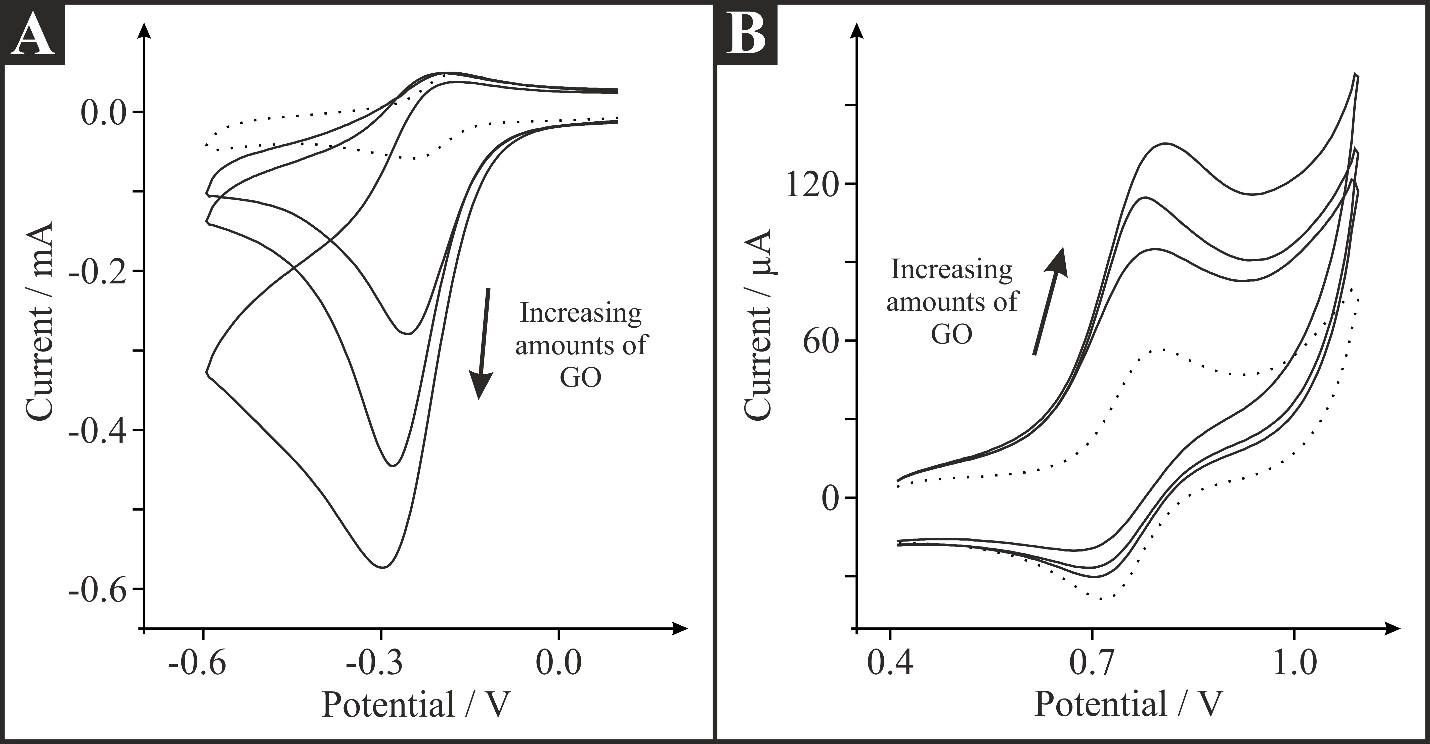


**ESI Figure S8**

Cyclic voltammetric responses of an EPPG electrode recorded in 1 mM TMPD (pH 7 PBS/0.1 M KCl) at a range of ‘slow’ scan rates: 2–15 mVs^–1^ (*vs*. SCE).


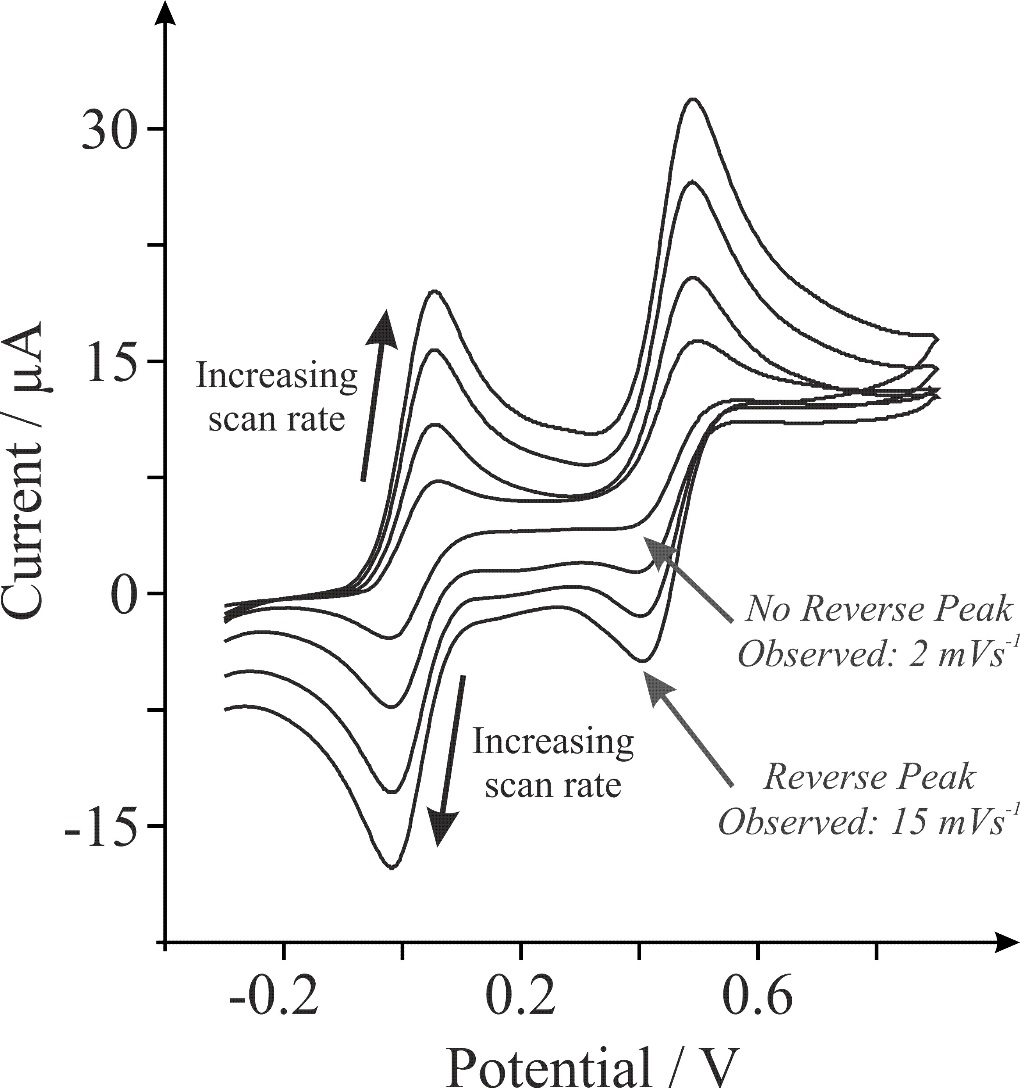


**References**

1. <http://www.specs.de/cms/front_content.php?idcat=209>, Accessed 18/04/2017.

2. M. P. Seah and S. J. Spencer, *J. Electron. Spectrosc. Relat. Phenom.*, 2006, 151, 178-181.

3. J. H. Scofield, *J. Electron. Spectrosc. Relat. Phenom.*, 1976, 8, 129-137.

4. <http://www.casaxps.com/>, Accessed 18/04/2017.

5. [www.graphene-supermarket.com](http://www.graphene-supermarket.com), Accessed 18/04/2017.

6. W. S. Hummers and R. E. Offeman, *J. Am. Chem. Soc.*, 1958, 80, 1339-1339.

7. D. R. Dreyer, S. Park, C. W. Bielawski and R. S. Ruoff, *Chem. Soc. Rev.*, 2010, 39, 228-240.

8. D. A. C. Brownson, A. C. Lacombe, M. Gómez-Mingot and C. E. Banks, *RSC Adv.*, 2012, 2, 665-668.

9. A. A. K. King, B. R. Davies, N. Noorbehesht, P. Newman, T. L. Church, A. T. Harris, J. M. Razal and A. I. Minett, *Sci. Rep.*, 2016, 6, 19491.

10. K. N. Kudin, B. Ozbas, H. C. Schniepp, R. K. Prud'homme, I. A. Aksay and R. Car, *Nano Lett.*, 2008, 8, 36-41.

11. A. R. Chowdhuri, S. Tripathy, S. Chandra, S. Roy and S. K. Sahu, *RSC Adv.*, 2015, 5, 49420-49428.

12. A. Dato, V. Radmilovic, Z. Lee, J. Phillips and M. Frenklach, *Nano Lett.*, 2008, 8, 2012-2016.

13. A. Dato, Z. Lee, K.-J. Jeon, R. Erni, V. Radmilovic, T. J. Richardson and M. Frenklach, *Chem. Commun.*, 2009, 40, 6095-6097.

14. Z. Lee, K.-J. Jeon, A. Dato, R. Erni, T. J. Richardson, M. Frenklach and V. Radmilovic, *Nano Lett.*, 2009, 9, 3365-3369.

15. D. A. C. Brownson, D. K. Kampouris and C. E. Banks, *Chem. Soc. Rev.*, 2012, 41, 6944-6976.

16. D. A. C. Brownson, S. A. Varey, F. Hussain, S. J. Haigh and C. E. Banks, *Nanoscale*, 2014, 6, 1607-1621.
